# Intelligent Programmable Membrane Nanosponge for Early Virus Blocking

**DOI:** 10.1002/advs.77592

**Published:** 2026-09-16

**Authors:** Ze Chen, Zhuojun He, Huimin Guo, Min Shi, Ruijing Liang, Lanlan Liu, Yeneng Dai, Qi Zhao, Ke Liu, Yang Zhou, Yang Zhang, Jian Ren, Bin Ju, Quan Fang, Zhaozhen Li, Pengfei Zhao, Mingbin Zheng, Lintao Cai

**Affiliations:** ^1^ Guangdong Key Laboratory of Nanomedicine CAS‐HK Joint Lab of Biomaterials Institute of Biomedicine and Biotechnology Shenzhen Institutes of Advanced Technology (SIAT) Chinese Academy of Sciences Shenzhen China; ^2^ National Clinical Research Center for Infectious Disease Shenzhen Third People's Hospital the Second Affiliated Hospital Southern University of Science and Technology Shenzhen China; ^3^ Cancer Center Institute of Translational Medicine Faculty of Health Sciences University of Macau Taipa Macau China; ^4^ The Affiliated Dongguan Songshan Lake Central Hospital Guangdong Medical University Dongguan China; ^5^ Sino‐European Center of Biomedicine and Health Shenzhen China

**Keywords:** competitive binding, endocytosis inhibition, infection early blockade, nanosponges, smart programmable membrane engineering

## Abstract

Coronaviruses, including severe acute respiratory syndrome coronavirus 2 (SARS‐CoV‐2), severe acute respiratory syndrome coronavirus (SARS‐CoV), and human coronavirus NL63 (HCoV‐NL63), infect host cells through spike (S) protein binding to angiotensin‐converting enzyme 2 (ACE2). Although soluble ACE2 can neutralize virions, its efficacy is limited by viral load and binding affinity. Herein, we engineered smart, programmable membrane‐engineered nanosponges (ACNPs) displaying high‐density ACE2 and encapsulating the viral entry inhibitor camostat. Combining customized ACE2 receptors with camostat enforces sequential recognition and endocytosis blockade: viruses are captured through S protein–ACE2 binding, followed by suppression of S protein cleavage to inhibit viral endocytosis and achieve ultra‐early blockade at the infection source. ACNPs exhibit an IC_50_ that is 22‐fold lower than that of ACE2‐only nanosponges and prevent more than 99% of viral entry. After intratracheal nebulization, ACNPs persist in the lungs for over 72 h, achieving over 92% viral clearance and 80%–100% survival. ACNPs also inhibit authentic HCoV‐NL63 infection, offering a deployable, non‐invasive, universal strategy for early intervention against pan ACE2‐dependent coronaviruses and future “X viruses”.

## Graphical abstract

A smart programmable nanosponge (ACNPs) displays high‐density viral receptors on its membrane and encapsulates fusion inhibitors to capture virions at the infection source through competitive binding and blocks their entry through protease inhibition for ultra‐early cascade interception. This modular, mucus‐penetrating platform shifts antiviral defence from reactive treatment to proactive, broad‐spectrum protection, offering a universal plug‐and‐play strategy for rapidly neutralizing known coronaviruses and preempting emerging viral threats (Created with BioRender.com).

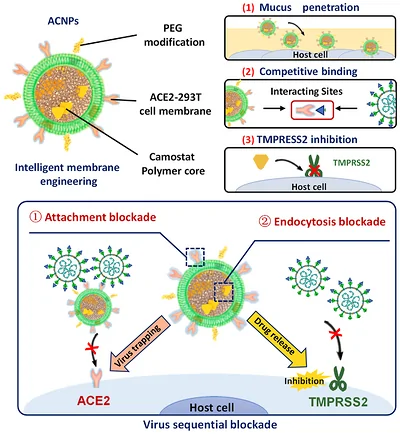

## 1 Introduction

Coronaviruses are highly mutable and transmissible RNA viruses in the Coronaviridae family [1, 2, 3]. Recurrent outbreaks of pan ACE2‐dependent coronaviruses, including severe acute respiratory syndrome coronavirus 2 (SARS‐CoV‐2), severe acute respiratory syndrome coronavirus (SARS‐CoV), and human coronavirus NL63 (HCoV‐NL63), have spread readily between individuals, causing acute respiratory distress, and significant mortality [4, 5]. These viruses primarily enter host cells through binding between the surface spike (S) protein and the angiotensin‐converting enzyme 2 (ACE2) receptor [6, 7, 8]. Specifically, the S1 subunit of the S protein binds host cell receptors, mediating viral attachment to the cell surface, and initiating entry [9, 10]. Various approaches have been developed to block coronavirus infection by disrupting S protein–ACE2 interactions, including ACE2 nanobodies and recombinant ACE2 proteins [11, 12, 13]. Recombinant soluble ACE2 can neutralize viruses, alleviate weight loss and lung pathology, and improve survival in preclinical models [14, 15]. Although soluble ACE2 or ACE2‐Ig competitively binds the S protein to block infection, their neutralization efficiency is constrained by fluctuating viral loads, insufficient competitive binding, and short in vivo half‐lives, making comprehensive viral blockade via ACE2‐mediated viral capture difficult to achieve [16, 17].

Blocking the entry of coronaviruses into host cells is crucial. Unneutralized viruses can invade cells via membrane fusion. After the viral S protein binds host ACE2, host serine proteases cleave the S protein, exposing the internal fusion peptide, and triggering fusion between viral and host membranes [9, 18]. This process ultimately leads to increased genome replication and virion assembly, potentially facilitating the accumulation of mutations during transmission, thereby driving adaptive evolution. Accordingly, S protein cleavage by host proteases such as transmembrane protease serine 2 (TMPRSS2) is required to expose the fusion peptide in the S2 subunit and trigger the conformational changes required for membrane fusion [19, 20]. Serine protease inhibitors, such as camostat, can effectively suppress S protein cleavage, markedly reducing SARS‐CoV‐2 cellular entry and lowering intracellular viral RNA levels by more than 100‐fold [10, 21, 22]. Therefore, combining viral neutralization with inhibition of viral endocytosis disrupts earlier stages of infection and offers an effective strategy to intercept viral invasion at its source [23, 24].

**FIGURE 1:**
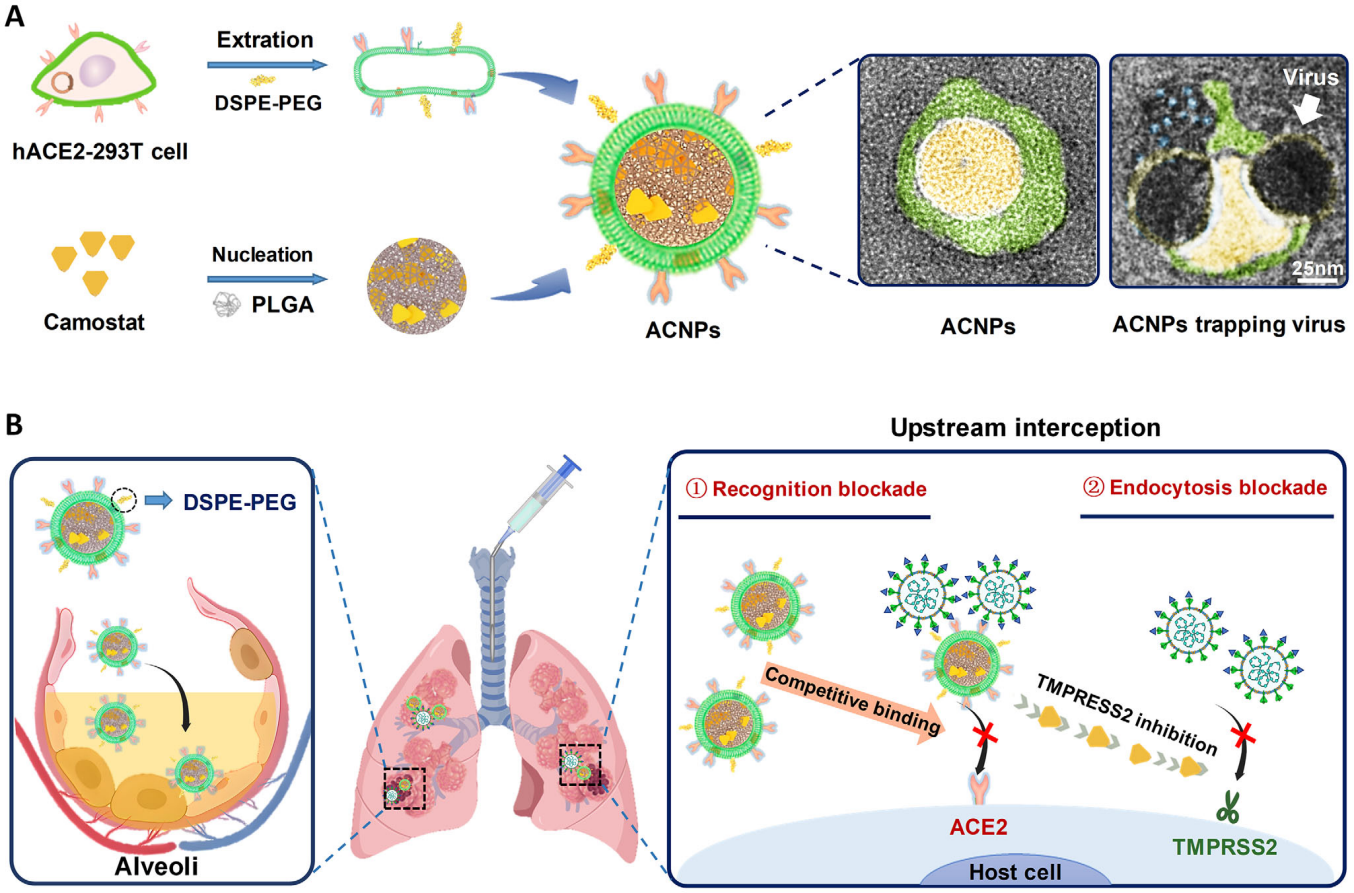
Schematic of mucus‐penetrating nanosponges (ACNPs) that neutralize virions and block viral entry, enabling ultra‐early source‐level suppression of pan ACE2‐dependent coronavirus infections. (A) Preparation of ACNPs and transmission electron microscopy (TEM) images showing ACNPs trapping virus. Human ACE2‐expressing 293T cell membranes were functionalized with DSPE‐PEG and coated onto camostat‐loaded polymeric cores to create ACNPs. These programmable nanosponges leverage membrane‐bound ACE2 for competitive viral neutralization and camostat to prevent viral internalization. (B) After administration, ACNPs penetrate mucus, and progressively block coronavirus infection by inhibiting viral recognition and endocytosis. (Created with BioRender.com).

In this study, we engineered inhalable smart programmable membrane‐engineered nanosponges (ACNPs) that neutralize virions and block endocytosis as a pan ACE2‐dependent coronavirus therapeutic strategy. ACNPs integrate high‐density ACE2 and DSPE‐PEG on the membrane to enable viral capture and mucus penetration, forming the outer shell around PLGA cores loaded with the viral entry inhibitor camostat. After administration, ACNPs penetrate mucus to block coronavirus infection by inhibiting viral recognition and endocytosis (Figure 1A,B). We characterized ACE2‐mediated binding between ACNPs and the SARS‐CoV‐2 S protein, assessed inhibition of TMPRSS2‐driven membrane fusion, and evaluated virion neutralization and viral‐entry blockade in vitro. In ACE2 mice challenged intranasally with pseudotyped or authentic SARS‐CoV‐2 (wild‐type, Delta, and Omicron EG.5) or HCoV‐NL63, ACNPs persisted in the lungs for over 72 h, efficiently cleared highly virulent or transmissible coronaviruses, improved survival, and mitigated lung pathology. Together, these findings demonstrate that combining competitive neutralization with membrane‐fusion inhibition enables broad and effective blockade of pan ACE2‐dependent coronavirus infections and provides a practical strategy for inhibiting viral transmission.

## 2 Results

### 2.1 Synthesis and Characterization of Cascade‐Blocking Antiviral Nanosponges (ACNPs)

**FIGURE 2:**
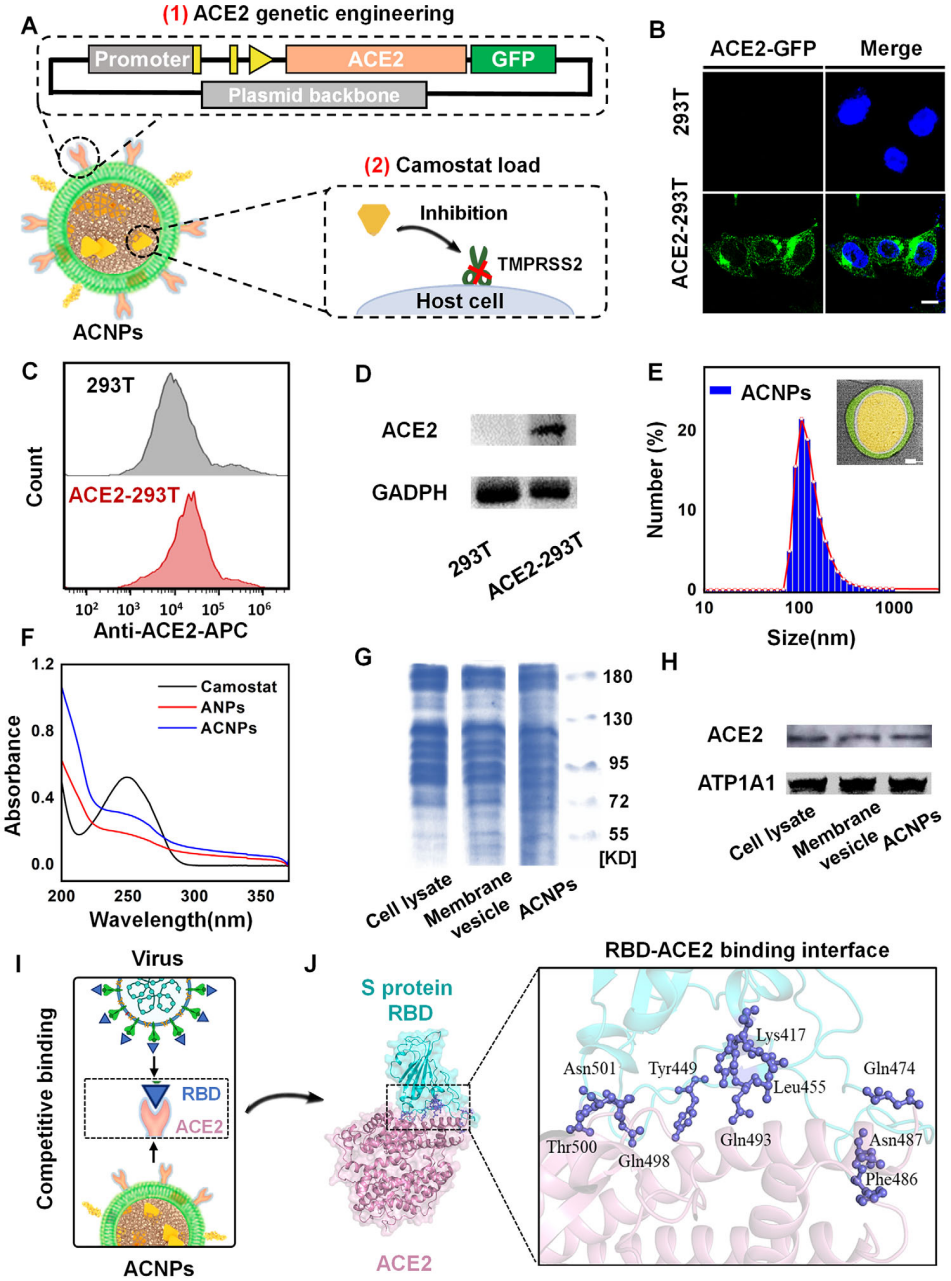
Characterization of cascade‐blocking antiviral nanosponges (ACNPs). (A) Schematic of ACNPs structure. ACE2 genetic engineering was utilized to acquire ACE2‐expressing membranes for viral neutralization, while encapsulated camostat inhibited TMPRSS2 (Created with BioRender.com). (B–D) Immunofluorescence imaging, flow cytometry, and western blotting of ACE2 expression in parental and engineered 293T cells. (E) Size distribution and transmission electron microscopy (TEM) image of ACNPs. (F) Absorption spectra of ACNPs. (G) SDS‒PAGE analysis of cell lysates, membrane vesicles, and ACNPs. (H) Western blotting analysis of specific membrane protein markers, including ACE2 and ATP1A1. (I, J) Schematic and molecular dynamics (MD) simulation of ACE2–RBD (receptor binding domain of the S protein) binding interfaces; purple: amino acid residues, blue: RBD binding sites, red: ACE2. (Panel I was created with Biorender.com. Panel J was generated using PyMOL 3.0.3).

To neutralize virions and block viral entry, we prepared mucus‐penetrating ACNPs comprising camostat‐loaded polymeric cores coated with ACE2‐expressing membranes derived from genetically engineered 293T cells (Figure 2A). Surface ACE2 on ACNPs competitively binds coronavirus particles, whereas camostat inhibits TMPRSS2‐mediated coronavirus transmembrane fusion with host cells [10, 21].

Immunofluorescence imaging, flow cytometry, and western blotting confirmed high ACE2 expression in genetically engineered 293T cells (ACE2‐293T; Figure 2B–D). After ACNPs preparation, dynamic light scattering (DLS) and transmission electron microscopy (TEM) revealed that the ACNPs were approximately 122 nm in diameter, with a typical core–membrane structure and good size stability (Figure 2E and Figure S1A). ACNPs also displayed a camostat absorption peak at 265 nm, confirming successful encapsulation with a 16.1% drug‐loading capacity (Figure 2F). As a control, ACE2‐expressing nanosponges without camostat (ANPs) were prepared using the same procedure. In vitro release assays showed that ACNPs released 41.5% of camostat within 24 h and 98% within 72 h (Figure S1B).

Protein gel electrophoresis and western blotting revealed that ACNPs retained protein profiles similar to those of ACE2‐293T cell lysates and displayed high ACE2 density (Figure 2G,H), indicating inheritance of the key SARS‐CoV‐2 binding receptor. The results demonstrated that ACNPs inherited this critical receptor protein (ACE2) from ACE2‐293T cells, which was essential for SARS‐CoV‐2 binding. To further evaluate the biosafety of the cell membrane‐derived formulation, residual cellular contaminants were examined. Residual DNA was markedly reduced after ACNPs purification, and SV40 large T antigen was substantially depleted in purified ACNPs (Figure S2).

Molecular dynamics (MD) simulations were used to evaluate binding between the viral S protein receptor‐binding domain (RBD) and host cell ACE2. Multiple residues, including Lys417, Tyr449, Leu455, Gln474, Asn487, Phe486, Gln493, Gln498, Thr500, and Asn501, were critical for ACE2–RBD binding (Figure 2I,J). These residues form hydrogen bonds and hydrophobic interactions that support high‐affinity binding between ACNPs and coronaviruses, enabling efficient viral capture. Structural analysis and molecular mechanics/generalized Born surface area (MM‐GBSA) calculations demonstrated that ACE2 maintained compatible binding interfaces with the WT, Delta, and Omicron EG.5 RBDs, with calculated binding free energies of −58.8, −60.3, and −53.6 kcal mol^−1^, respectively. The results indicated stable ACE2–RBD interactions across the three variants and support the broad variant‐recognition capability of ACNPs (Figure S3). This interaction immobilizes virions and limits dissemination, creating a robust barrier to viral propagation.

Given that virus‐infected lungs are filled with mucus that impedes drug penetration [25, 26], we assessed the mucus‐penetrating ability of ACNPs with and without PEG modification. DSPE‐PEG–functionalized ACNPs traversed the mucus layer and promoted viral capture in mucus‐rich alveolar spaces (Figure S4) [27, 28, 29]. By combining competitive viral binding with entry blockade, this modular pan ACE2‐dependent coronavirus platform shows strong potential for broad‐spectrum antiviral activity.

### 2.2 ACNPs Competitively Bind SARS‐CoV‐2 and Inhibit Viral Entry Into Cells

**FIGURE 3:**
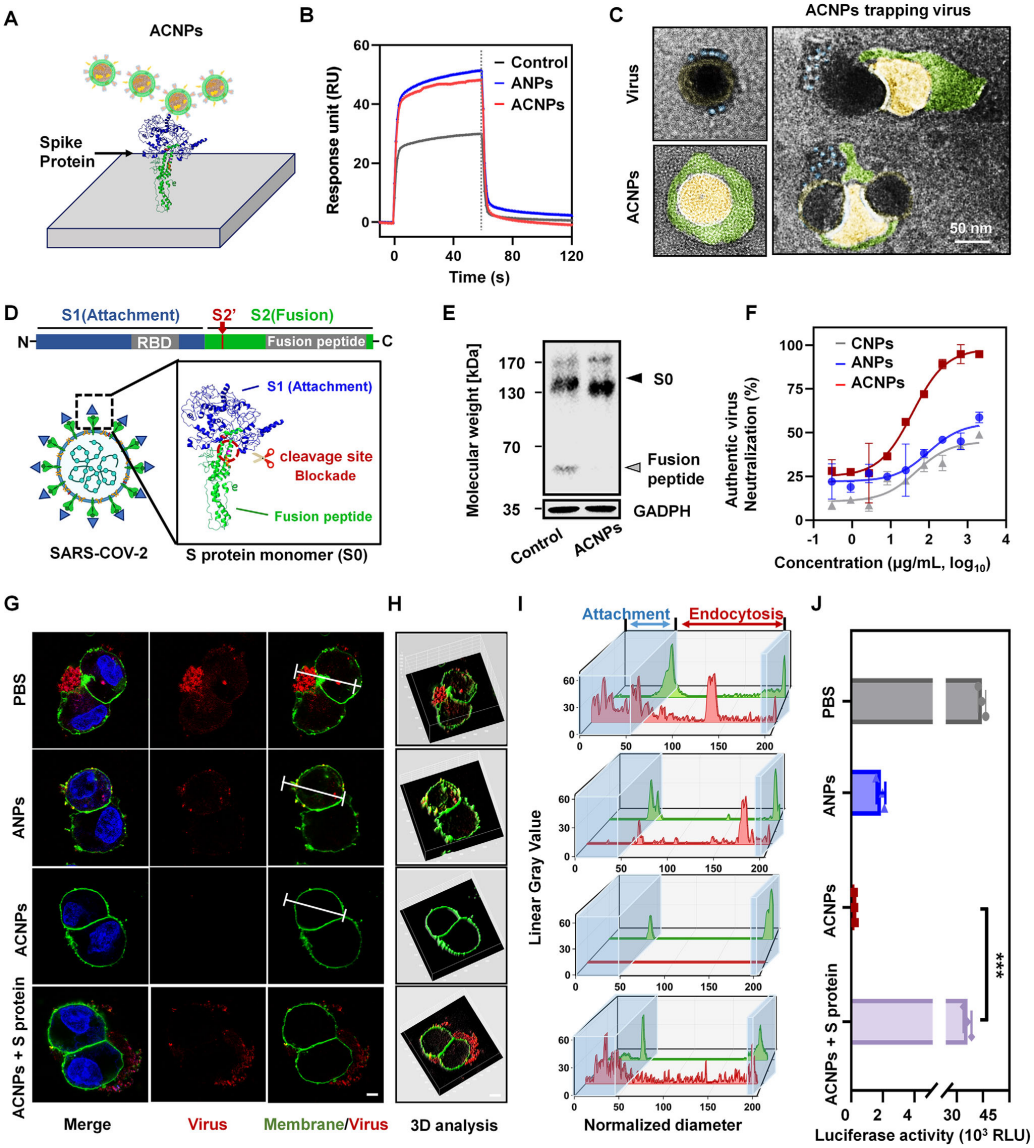
Early ACNPs intervention blocks coronavirus infection by inhibiting viral recognition and endocytosis. (A) Surface plasmon resonance (SPR) assay workflow. S protein was immobilized on a sensor chip, and ACNPs binding affinity for SARS‐CoV‐2 was measured. (B) SPR‐determined binding affinity of ANPs and ACNPs with SARS‐CoV‐2. (C) Transmission electron microscopy (TEM) images of virus‐trapped ACNPs. (D) Structural depiction of the SARS‐CoV‐2 spike protein monomer (S0). After host–cell ACE2 binds the viral S1 subunit, TMPRSS2 cleaves S0 and exposes the fusion peptide, enabling membrane fusion. (E) Western blotting showing that ACNPs inhibit spike‐protein cleavage and prevent fusion peptide exposure. (F) Blockade of authentic wild‐type SARS‐CoV‐2 infection by CNPs, ANPs, and ACNPs in Calu‐3 cells (n = 3). (G) Confocal images showing that ACNPs block both SARS‐CoV‐2 binding and endocytosis (scale bar, 3 µm). (H) Three‐dimensional imaging of viral signals in Calu‐3 cells. (I) Fluorescence intensity analysis of the underlined region in (G). (J) Bioluminescence quantification of SARS‐CoV‐2 inhibition into host cells. Data are presented as mean ± SD (*n* = 3). Significance is calculated using two‐tailed Student's *t*‐test. *** *p* < 0.001. Panel A and D are created with Biorender.com.

Given that ACNPs display abundant ACE2, they can mimic host cells and efficiently bind coronaviruses. To assess their affinity for the viral S protein, the S protein was immobilized on a sensor chip to capture nanoparticles, and surface plasmon resonance (SPR) was used to evaluate binding capacity (Figure 3A). Both ANPs and ACNPs strongly bound the S protein chip, exhibiting a rapid increase in response units (RUs) that indicated high binding affinity and efficient viral capture. Kinetic fitting analysis further revealed apparent equilibrium dissociation constant (K_D_) values of 0.321 and 0.323 nm for ANPs and ACNPs, respectively. In contrast, nanoparticles lacking ACE2, as control, showed only weak binding (K_D_ = 9.433 nm). The affinities of ANPs and ACNPs were also much stronger than soluble ACE2 (K_D_ = 4.7 nm), indicating that the multivalent presentation of ACE2 on nanoparticle surface substantially enhanced binding affinity (Figure 3B and Table S1). TEM images further revealed that ACNPs captured coronaviruses through sponge‐like deformation (Figure 3C). After binding, ACNPs trapped the virus and formed a physical barrier that shielded viral active sites, preventing interaction with host cell receptors such as ACE2 and thereby inhibiting attachment and entry. Thus, as plug‐and‐play functional nanosponges, ACNPs showed strong virion‐trapping capacity, and neutralized viral invasion.

Despite their high affinity for SARS‐CoV‐2, a small fraction of virus may evade competitive capture and attach to host cells through S protein–ACE2 interactions. The S0 monomer is then cleaved at the S2’ site by TMPRSS2 on the host‐cell surface, exposing the fusion peptide and promoting viral–host membrane fusion (Figure 3D). Camostat‐loaded ACNPs blocked S0 cleavage, preventing fusion peptide exposure, and membrane fusion (Figure 3D,E). Quantitatively, camostat‐loaded PLGA nanoparticles without membrane coating (CNPs) achieved 32% pseudovirus inhibition, consistent with camostat‐mediated TMPRSS2 inhibition. ANPs markedly enhanced viral neutralization via ACE2‐mediated viral capture. Owing to combinational ACE2‐mediated viral capture and camostat‐mediated TMPRSS2 inhibition, ACNPs achieved 99% inhibition (Figure S5). In a plaque‐reduction neutralization assay using authentic wild‐type SARS‐CoV‐2, CNPs exhibited camostat‐mediated cell entry inhibition and antiviral activity, with an IC_50_ of 2265 µg/mL. ANPs could capture coronaviruses due to competitive ACE2‐S protein binding, and achieved an IC_50_ of 822 µg/mL to SARS‐CoV‐2 virus. Notably, leveraging virus capture and endocytosis inhibition cascade blockade, ACNPs profoundly augmented antiviral activity, reducing the IC_50_ to merely 38 µg/mL. This improvement underscored the superior potency of ACNPs in neutralizing SARS‐CoV‐2 (Figure 3F).

SARS‐CoV‐2 produced abundant intracellular and extracellular viral signals, whereas ANPs reduced extracellular virus through competitive binding but did not fully prevent internalization. In contrast, ACNPs‐treated cells showed no detectable viral signals within or around cells, indicating dual blockade of viral attachment and endocytosis. Pre‐blocking ACNPs with S protein significantly increased extracellular viral signals and pseudoviral load, providing direct cellular evidence that ACNPs inhibited viral binding to host cells through ACE2‐mediated competitive capture (Figure 3G). Quantitative line scanning and 3D reconstruction confirmed the absence of intracellular viral signals after ACNPs treatment (Figure 3H,I). A luciferase assay demonstrated that ACNPs (1 mg/mL) reduced wild‐type SARS‐CoV‐2 pseudovirus luminescence by nearly 200‐fold, indicating highly efficient viral inhibition (Figure 3J and Figure S5).

Overall, ACNPs competitively bind viral S proteins through ACE2‐expressing surfaces and inhibit TMPRSS2‐mediated membrane fusion. This cascade mechanism disrupts two key steps in viral entry—receptor recognition and protease‐dependent membrane fusion—achieving potent antiviral activity through active functional programming rather than passive biomimicry.

### 2.3 ACNPs Block Pseudotyped SARS‐CoV‐2 Infection In Vivo

**FIGURE 4:**
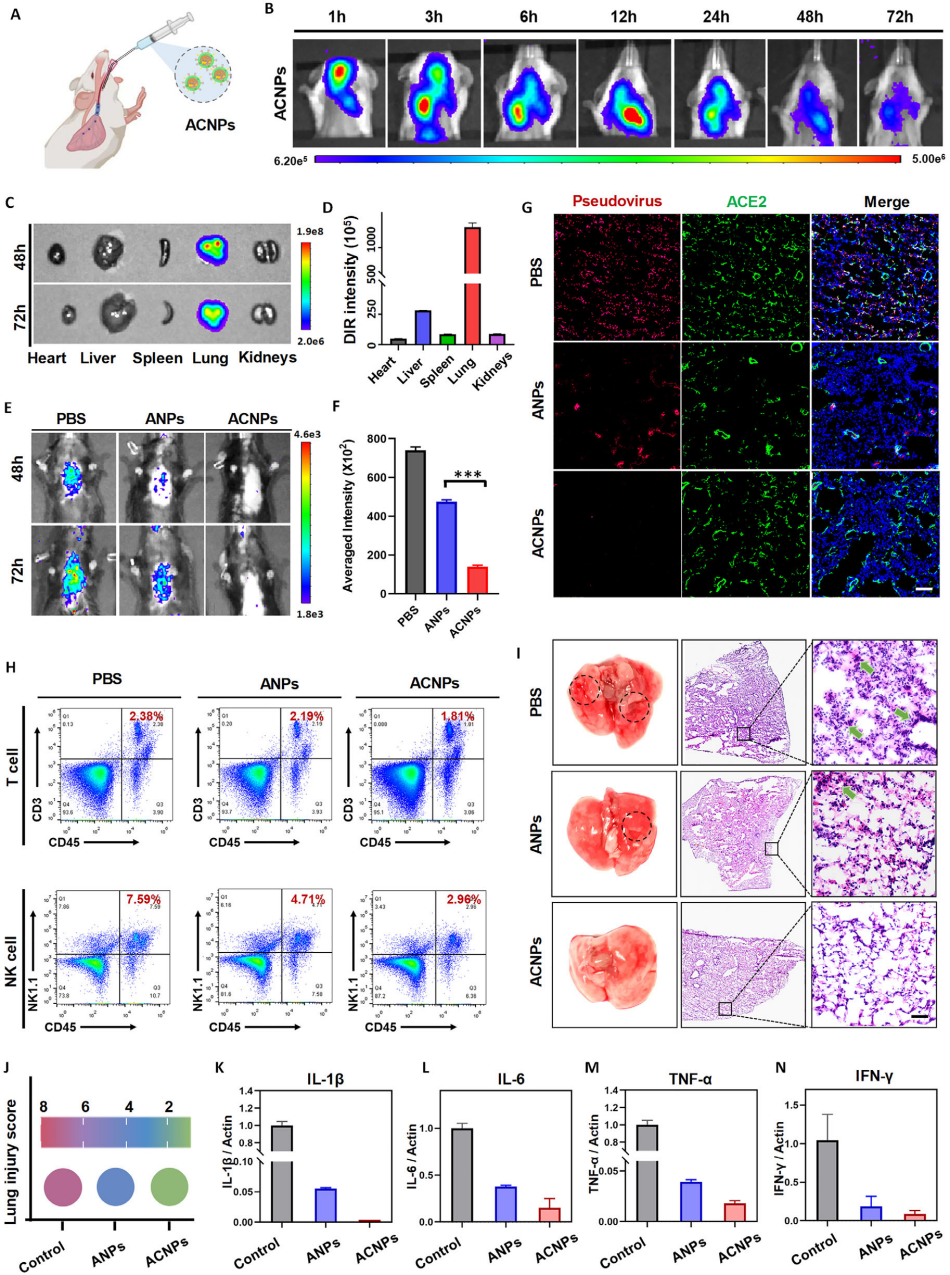
Inhibition of SARS‐CoV‐2 wild‐type pseudovirus infection in ACE2‐expressing mice. (A) Schematic of tracheal atomization administration (Created with BioRender.com). (B) Time‐lapse in vivo near‐infrared (NIR) images of ACE2‐expressing mice after ACNPs administration. (C) Ex vivo fluorescence (FL) images of major organs 48 and 72 h after ACNPs administration. (D) Quantitative biodistribution of ACNPs in major organs at 48 and 72 h. (E) Viral bioluminescence images in pseudovirus‐infected ACE2 mice at 48 and 72 h post‐treatment. (F) Quantification of retained viral bioluminescence in vivo after treatment. (G) Immunofluorescence image of transverse lung sections; scale bar, 50 µm. (H) Flow cytometry analysis of T cells and NK cells collected from mouse lungs after treatment. (I, J) H&E staining and pathological scoring of lungs after treatment; green arrow: inflammatory cell infiltration; scale bar, 50 µm. (K–N) Pulmonary cytokine levels (IL‐1β, IL‐6, TNF‐α, and IFN‐γ) analyzed by qPCR. Data are presented as mean ± SD.

We next evaluated the in vivo antiviral efficacy of ACNPs in ACE2‐expressing mice with lung infection using non‐invasive inhalation via a portable nebulizer (Figure 4A). To examine pulmonary retention, DiR‐labeled ACNPs were administered by inhalation and tracked using an in vivo imaging system (IVIS). ACNPs remained detectable in the lungs for up to 72 h after a single inhaled dose (Figure 4B–D). Across the assessed time points, lung tissue exhibited the strongest ACNPs fluorescence signal among major organs, and ACNPs were broadly distributed throughout the lungs. Quantitative analysis further showed that pulmonary ACE2 levels retained approximately 87.8%, 42.0%, 34.7%, and 19.6% of the initial level at 24, 48, 72, and 96 h, respectively. Over the same period, approximately 74.0%, 43.2%, 21.2%, and 12.0% of the camostat remained in the lungs. Both components remained detectable over 96 h, confirming the prolonged pulmonary retention of ACNPs (Figure S6). This extensive and sustained pulmonary retention supports ACNPs as a promising long‐acting strategy for viral intervention, potentially reducing the need for frequent dosing.

To assess antiviral efficacy, K18‐ACE2 transgenic mice were challenged with pseudotyped SARS‐CoV‐2 using a MicroSprayer Aerosolizer. Twenty min later, mice were randomly assigned to receive intratracheal treatment with ANPs, ACNPs, or phosphate‐buffered saline (PBS) as a control. Bioluminescence and immunofluorescence imaging confirmed strong antiviral activity by ACNPs in lung infection. Compared with the intense pseudovirus bioluminescence signal in control mice, ACNPs‐treated mice showed markedly reduced pulmonary pseudovirus signals, confirming significant inhibition of viral infection through cascade blockade of recognition and endocytosis (Figure 4E,F). Viral immunofluorescence staining further revealed undetectable pseudovirus signals in ACNPs‐treated lungs, confirming effective viral clearance (Figure 4G and Figure S7). In the dose‐response study, ACNPs containing 5 mg/mL camostat and 6 mg/mL membrane protein (C_1_) achieved approximately 92% pseudovirus inhibition, comparable to ACNPs containing 15 mg/mL camostat and 18 mg/mL membrane protein (3C_1_). Reducing the dose of ACNPs substantially decreased inhibition efficacy. Thus, ACNPs containing 5 mg/mL camostat and 6 mg/mL membrane protein were defined as the minimum effective dose (Figure S8).

Quantitative viral bioluminescence signals in vivo and immunofluorescence quantification of lung tissue sections indicated that a single‐dose treatment of ACNPs achieved effective and equivalent efficacy (over 90% viral inhibition) in comparison with three‐dose treatment (Figure S9). And three‐dose administrations caused no detectable histopathological abnormalities or significant changes in serum biochemical indicators, suggesting no evident cumulative systemic toxicity (Figure S10).

Moreover, administered at 24, 48, or 72 h after pseudovirus challenge, ACNPs reduced bioluminescence signals by approximately 73%, 54%, and 24%, and immunofluorescence signals by 95%, 71%, and 34%, respectively. It demonstrated that ACNPs were not limited to immediate post‐exposure intervention, but also effective for early‐stage infection treatment (Figure S11). ACNPs also exhibited enhanced antiviral efficacy in mice infected with Delta‐ and Omicron‐pseudotyped viruses, reducing the Delta, and Omicron bioluminescence signals by 92% and 95%, respectively (Figure S12). Together, these results demonstrated that sequential recognition and endocytosis blockade enable broad‐spectrum inhibition of pseudotyped SARS‐CoV‐2 variants in vivo.

Considering that pulmonary viral infection triggers immune‐cell recruitment and inflammatory injury, we next assessed immune infiltration of T cells and natural killer (NK) cells, which contribute to cytokine release, lung inflammation, and tissue damage (Figure 4H). Hematoxylin and eosin (H&E) staining of lung sections from infected control mice revealed severe lung injury, including thickened alveolar septa and inflammatory‐cell infiltration. In contrast, ACNPs‐treated mice showed markedly reduced immune‐cell infiltration and lung damage (Figure 4I,J). ACNPs treatment also significantly reduced pulmonary cytokine levels, including interleukin (IL)‐1β, IL‐6, tumor necrosis factor (TNF)‐α, and interferon (IFN)‐γ, compared with PBS or ANPs (Figure 4K–N). These histopathologic and inflammatory analyses demonstrated that ACNPs reduce viral load, limit immune‐cell infiltration, and protect against downstream pulmonary tissue injury.

### 2.4 Transcriptomic Profiling and Mechanistic Landscape of ACNPs‐Mediated Protection

**FIGURE 5:**
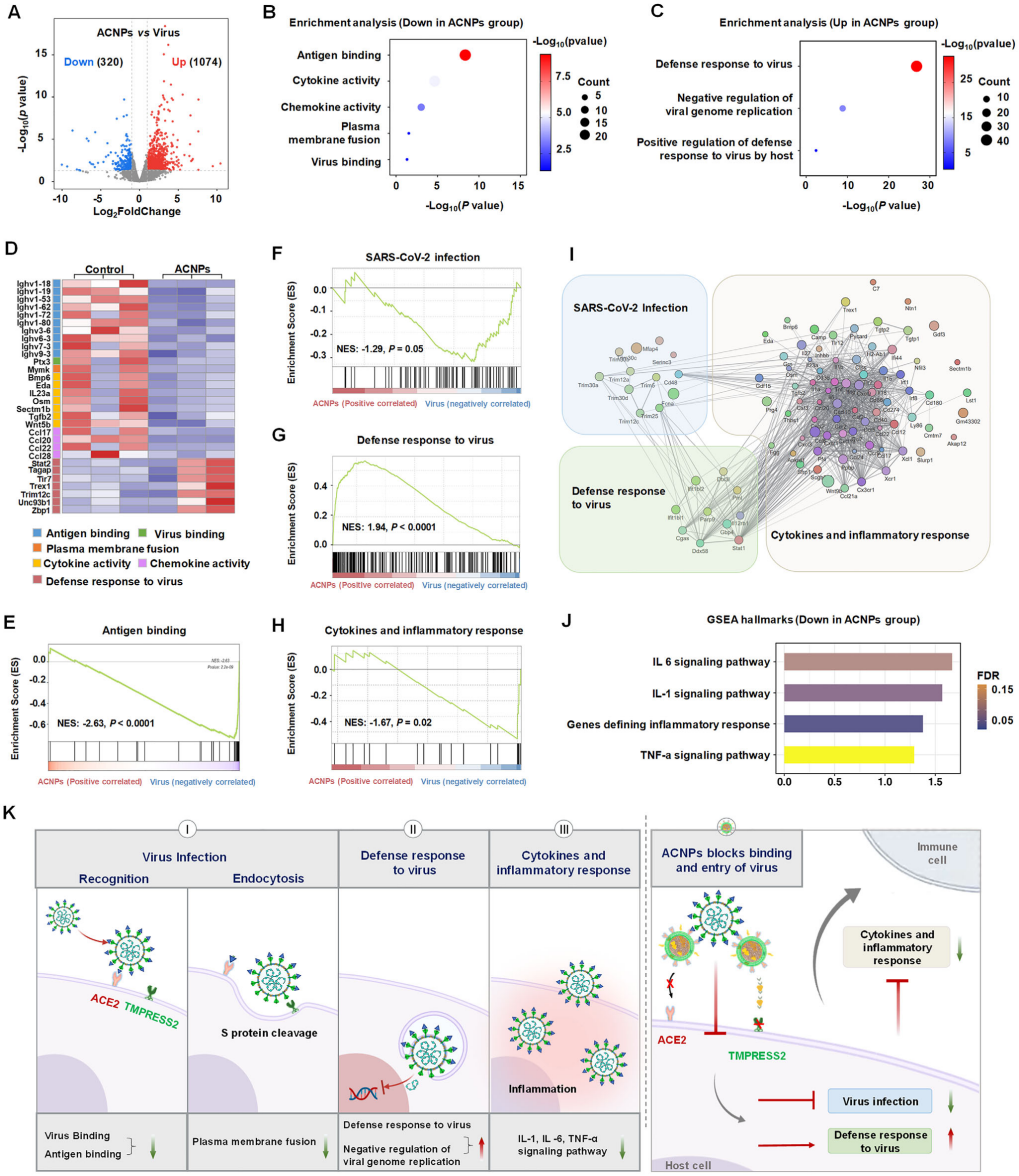
Transcriptomic analysis following ACNPs treatment of SARS‐CoV‐2 pseudovirus infection in ACE2‐expressing mice. (A) Volcano map of differential mRNA expression in lungs after ACNPs treatment. (B, C) Gene Ontology (GO) enrichment analysis of differentially expressed genes after ACNPs treatment. (D) Heatmap of genes associated with viral infection and inflammation; data are presented as mean ± SD (*n* = 3). (E–H) GSEA enrichment plots for antigen binding, SARS‐CoV‐2 infection, defense response to virus, and cytokine/inflammatory response. (I) Protein–protein interaction (PPI) network of regulators associated with SARS‐CoV‐2 infection, antiviral defense, and cytokine/inflammatory‐response pathways. (J) Downregulated GSEA hallmark pathways related to cytokine and inflammatory responses in the ACNPs group. NES, normalized enrichment score; FDR, false discovery rate. (K) Schematic of ACNPs‐mediated dual‐site blockade of SARS‐CoV‐2 entry (Created with BioRender.com). ACNPs simultaneously interfere with ACE2‐dependent viral recognition and TMPRSS2‐mediated spike priming, thereby preventing early viral binding, membrane fusion, and cellular entry while enhancing host antiviral defense and alleviating pulmonary inflammation.

To define the molecular mechanisms underlying ACNPs‐mediated protection, we performed RNA sequencing on lung tissues from pseudovirus‐infected mice after ACNPs treatment. Compared with virus‐infected controls, ACNPs treatment significantly altered the expression of 1394 genes, including 1074 upregulated and 320 downregulated genes (Figure 5A).

Gene Ontology (GO) enrichment analysis showed that ACNPs reduced the expression of genes associated with viral antigen binding, virus binding, viral membrane fusion into host cells, cytokine activity, and chemokine activity (Figure 5B). In parallel, ACNPs upregulated genes involved in antiviral defense and negative regulation of viral genome replication (Figure 5C). Consistent with the dual blockade of viral recognition and endocytosis, ACNPs markedly suppressed transcriptional programs linked to infection and lung inflammation. Heatmap analysis further showed that ACNPs treatment reversed infection‐induced gene expression patterns related to antigen binding, viral membrane fusion, and lung inflammation (cytokines and chemokines; Figure 5D).

Gene set enrichment analysis (GSEA) confirmed downregulation of antigen binding, SARS‐CoV‐2 infection, cytokine, and inflammatory response pathways, while antiviral defense pathways were markedly upregulated in lungs from ACNPs‐treated mice (Figure 5E–H). These transcriptomic shifts indicate that ACNPs reprogram the pulmonary immune landscape toward viral suppression and controlled inflammation.

Protein–protein interaction (PPI) network analysis provided further insight into ACNPs‐regulated host responses. The network contained three major functional clusters: SARS‐CoV‐2 Infection (light blue), comprising entry‐related factors such as MFAP4 and Trim family members; defense response (light green), including innate sensors such as CGAS, DDX58, and STAT1; and cytokine/inflammation signaling (beige), centered on mediators such as IL‐6 and CXCL10 (Figure 5I). Crosstalk among these modules indicated that ACNPs‐mediated dual‐site blockade of ACE2‐dependent recognition and TMPRSS2‐mediated spike priming not only disrupts viral entry but also enhances innate antiviral defense and alleviates inflammatory dysregulation. Hallmark GSEA further showed that ACNPs treatment significantly downregulated core pro‐inflammatory pathways, including IL‐6, IL‐1, TNF‐α, and inflammatory‐response gene sets (Figure 5J). Together, these data demonstrate that ACNPs suppress viral‐entry programs while restoring a more balanced pulmonary immune response.

We further evaluated the potential toxicity of inhaled ACNPs in mice. Histopathologic analysis showed that lung tissues after ACNPs administration had immune‐cell infiltration comparable to that of controls, with no evidence of lesion formation or tissue damage (Figure S13A). Hepatic function markers (Figure S13B), renal function markers (Figure S13C), and multiple blood parameters (Figure S13D,E) also remained within control ranges after ACNPs treatment. These findings demonstrate the excellent in vivo biocompatibility of ACNPs after inhalation. In summary, ACNPs achieve dual‐site blockade of SARS‐CoV‐2 entry by simultaneously disrupting ACE2‐dependent viral recognition and TMPRSS2‐mediated spike priming. This mechanism prevents early viral binding, membrane fusion, and cellular entry while strengthening antiviral defense and reducing pulmonary inflammation (Figure 5K).

### 2.5 Broad and Potent In Vivo Activity Against Authentic Wild‐Type, Delta, and Omicron SARS‐CoV‐2 Variants

**FIGURE 6:**
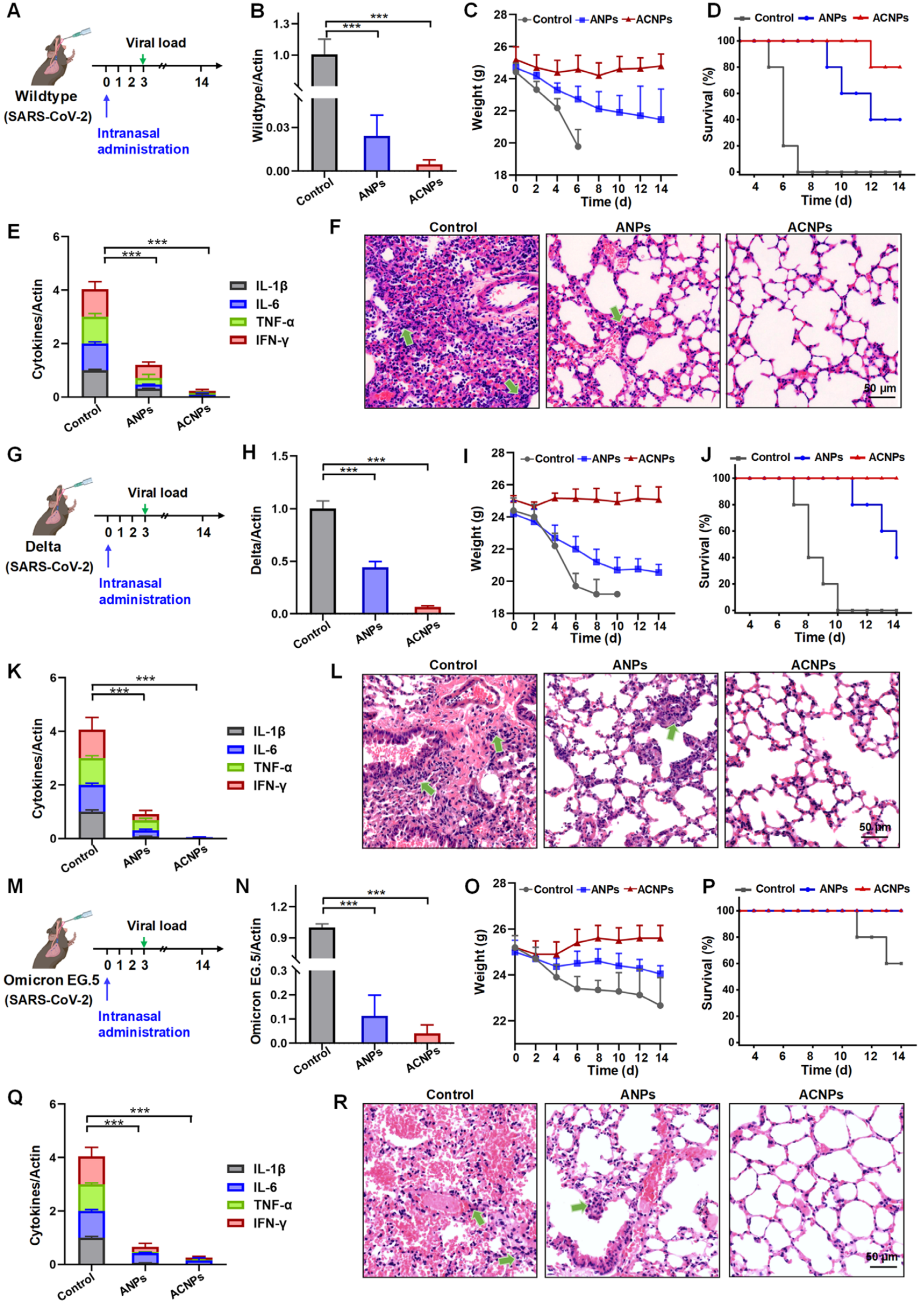
ACNPs inhibit infection by authentic wild‐type SARS‐CoV‐2 and the highly virulent Delta or highly transmissible Omicron variants in vivo. (A) Protocol for authentic wild‐type SARS‐CoV‐2 infection and ACNPs treatment in ACE2 mice (Created with BioRender.com). (B) Analysis of wild‐type viral loads in lungs after treatment. (C) Body weight change curves. (D) Survival curves. (E) Cytokine analysis in lungs. (F) H&E staining of representative lung sections after treatment; blue arrow: dense inflammatory cell infiltration. (G) Protocol for authentic Delta infection and ACNPs treatment in transgenic mice (Created with BioRender.com). (H) Analysis of Delta viral loads in lungs after treatment. (I) Body weight change curves. (J) Survival curves. (K) Cytokine analysis in the lung. (L) H&E staining of representative lung sections after treatment; green arrow: dense inflammatory cell infiltration. (M) Protocol for authentic Omicron EG.5 infection and ACNPs treatment in transgenic mice (Created with BioRender.com). (N) Analysis of Omicron EG.5 viral loads in lungs after treatment. (O) Body weight change curves. (P) Survival curves. (Q) Cytokine analysis in lungs. (R) H&E staining of representative lung sections after treatment; green arrow: dense inflammatory cell infiltration. All data are presented as mean ± SD (*n* = 3).

The continued emergence of highly virulent or transmissible SARS‐CoV‐2 variants underscores the need for broad‐spectrum antiviral strategies [30, 31]. We therefore evaluated ACNPs efficacy against authentic wild‐type SARS‐Cov‐2, Delta, and Omicron EG.5 in ACE2 mice. After intranasal challenge with wild‐type SARS‐CoV‐2, ACNPs treatment markedly reduced lung viral loads, achieving greater than 99% viral clearance (Figure 6A,B). Untreated control mice lost 18.9% of body weight by day 6 and reached 100% mortality, whereas ACNPs‐treated mice showed no significant weight loss and maintained 80% survival, with only one death on day 12 (Figure 6C,D).

Lung tissues collected at death or euthanasia on day 14 showed markedly elevated pro‐inflammatory cytokines (IL‐1β, IL‐6, TNF‐α, and IFN‐γ) in control mice (Figure 6E). Both ANPs and ACNPs significantly reduced inflammatory cytokine levels, with ACNPs producing the strongest effect and lowering cytokines by more than 95%. This potent antiviral activity was accompanied by improved lung pathology, including reduced alveolar septal thickening and inflammatory‐cell infiltration in ACNPs‐treated mice compared with untreated controls (Figure 6F).

We next examined whether ACNPs retained efficacy against globally dominant SARS‐CoV‐2 variants. In Delta‐infected mice, ACNPs suppressed lung viral replication and achieved more than 95% viral clearance (Figure 6G,H). ACNPs treatment also prevented weight loss, ensured 100% survival (Figure 6I,J), and significantly reduced pro‐inflammatory cytokine levels and lung injury (Figure 6K,L). Similarly, ACNPs showed strong antiviral activity against the highly transmissible Omicron EG.5 variant, significantly reducing lung viral loads (Figure 6M,N). ACNPs treatment protected against weight loss, achieved 100% survival (Figure 6O,P), reduced inflammatory cytokine levels, and alleviated lung pathology (Figure 6Q,R). Collectively, these data established ACNPs as potent broad‐spectrum antiviral agents capable of rapidly clearing diverse highly virulent or transmissible SARS‐CoV‐2 variants in vivo while reducing associated pulmonary inflammation and tissue injury.

### 2.6 ACNPs Block Authentic HCoV‐NL63 Infection in ACE2 Mice

Human coronavirus NL63 (HCoV‐NL63) circulates worldwide and primarily affects older adults, immunocompromised individuals, and infants, causing severe respiratory diseases, including acute respiratory distress syndrome (ARDS) [32, 33, 34, 35]. Similar to SARS‐CoV and SARS‐CoV‐2, HCoV‐NL63 initiates host infection through the ACE2 receptor, making it a suitable model for evaluating the broad‐spectrum efficacy of the ACE2‐targeting ACNPs platform. We therefore assessed ACNPs activity against HCoV‐NL63 infection in an ACE2 mouse model.

**FIGURE 7:**
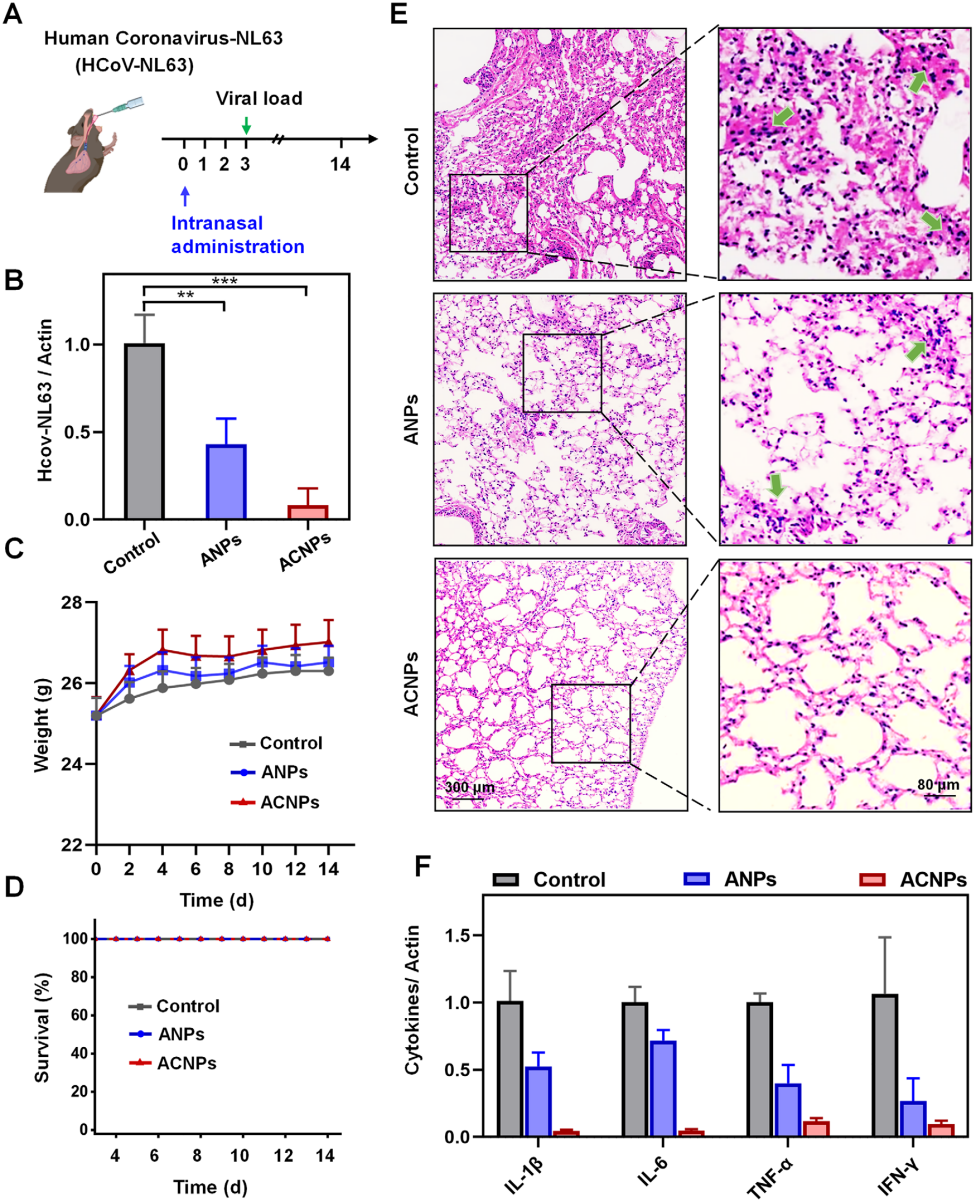
ACNPs inhibit infection by authentic HCoV‐NL63, an ACE2‐dependent epidemic coronavirus. (A) Protocol for authentic wild‐type HCoV‐NL63 infection and ACNPs treatment in ACE2 mice (Created with BioRender.com). (B) Analysis of HCoV‐NL63 viral loads in lungs after treatment. (C) Body weight change curves. (D) Survival curves. (E) H&E staining of representative lung sections after treatment; green arrow: dense inflammatory cell infiltration. (F) Cytokine analysis in lungs by qPCR. Data are presented as mean ± SD (*n* = 3). Significance was calculated using one‐way ANOVA with Tukey's post‐hoc test. *p < 0.05, **p < 0.01, ***p < 0.001.

Following intranasal challenge with ACE2‐dependent HCoV‐NL63, ACNPs treatment markedly reduced lung viral loads and achieved more than 93% viral clearance compared with infected controls (Figure 7A,B). Given that HCoV‐NL63 causes relatively mild, non‐lethal disease in this model, groups showed increased body weight and 100% survival; however, ACNPs‐treated mice exhibited a greater weight increase (Figure 7C,D). H&E staining further showed significantly reduced inflammatory cell infiltration in ACNPs‐treated lungs, whereas untreated infected mice displayed widespread inflammation and thickened alveolar septa (Figure 7E). ACNPs treatment also markedly reduced pulmonary pro‐inflammatory cytokines, including IL‐1β, IL‐6, TNF‐α, and IFN‐γ, compared with infected controls (Figure 7F).

By combining ACE2‐mediated virus binding with TMPRSS2‐dependent endocytosis inhibition, ACNPs overcame the limitations of pathogen‐specific therapies and function as a modular plug‐and‐play pan ACE2‐dependent coronavirus therapeutic platform. This programmable strategy could shift global antiviral responses from reactive countermeasures toward proactive, preemptive defense against current and future coronavirus‐driven epidemics and pandemics.

### 2.7 Long‐Term Biosafety of Repeated ACNPs Administration

**FIGURE 8:**
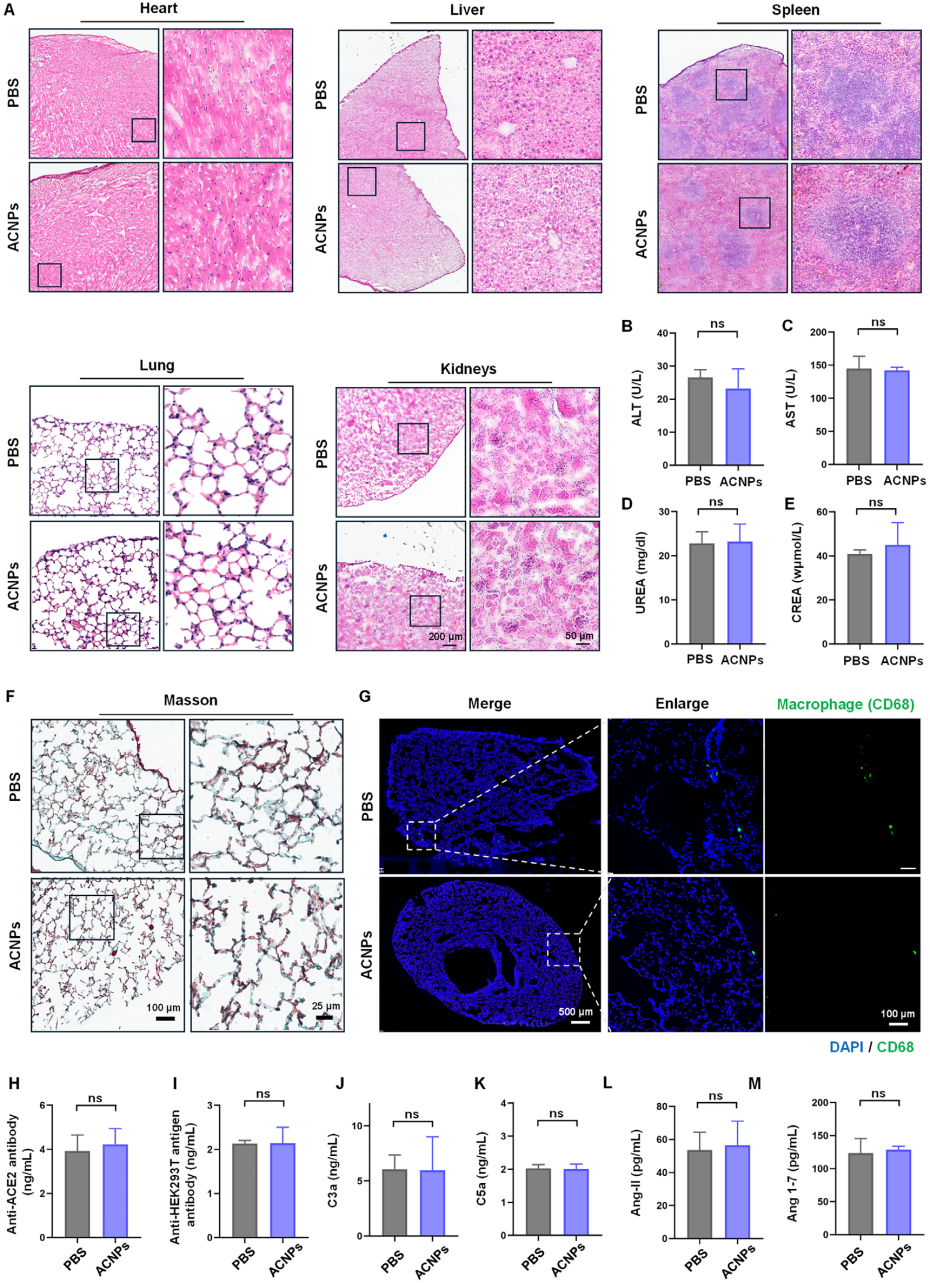
Long‐term biosafety evaluation of repeated ACNPs administration. Mice received ACNPs three times weekly for 28 days, after which major organs, lung tissues, serum, and plasma were collected for analysis. (A) Representative H&E‐stained sections of the heart, liver, spleen, lungs, and kidneys from control and ACNPs‐treated mice. Boxed regions are shown at higher magnification. Scale bar, 50 µm. (B–E) Serum levels of alanine aminotransferase (ALT), aspartate aminotransferase (AST), urea (UREA), and creatinine (CREA). (F) Representative Masson's trichrome staining of lung sections showing collagen deposition and pulmonary morphology. Boxed regions are shown at higher magnification. Scale bars, 100 and 25 µm. (G) Representative immunofluorescence images of lung sections stained for the macrophage marker CD68 (green) and nuclei (DAPI, blue). Boxed regions are enlarged in the middle panels. Scale bars, 500 and 100 µm. (H–K) Serum levels of anti‐ACE2 antibodies, anti‐HEK293T membrane antigen antibodies, complement component C3a, and complement component C5a, respectively. (L, M) Serum levels of angiotensin II (Ang II) and angiotensin 1–7 (Ang 1–7) in mice after repeated ACNPs administration. Data are presented as the mean ± SD. Statistical significance is determined using Student's *t*‐test; ns indicates not significant; ***p < 0.001.

To evaluate the long‐term biosafety of repeated ACNPs administration, healthy mice received ACNPs three times weekly for 28 days. H&E staining revealed no apparent histopathological abnormalities, inflammatory infiltration, or structural damage in the heart, liver, spleen, lungs, or kidneys compared with the control group (Figure 8A). Consistently, serum alanine aminotransferase (ALT), aspartate aminotransferase (AST), urea (UREA), and creatinine (CREA) levels remained unchanged, indicating no detectable hepatic or renal toxicity (Figure 8B–E). Further pulmonary evaluation showed no obvious collagen deposition, fibrotic lesions, or disruption of alveolar architecture by Masson's trichrome staining, while CD68 immunofluorescence revealed no evident macrophage infiltration in the lungs (Figure 8F,G). Repeated ACNPs administration also did not alter serum anti‐ACE2 antibody, anti‐HEK293T membrane antigen antibody, C3a, or C5a levels, suggesting no detectable humoral immune response or complement activation (Figure 8H–K).

To evaluate the impact of repeated ACNPs administration on ACE2‐associated physiological functions, systemic renin–angiotensin system (RAS) homeostasis was assessed after 28 days of treatment. Serum Ang II and Ang 1–7 levels showed no significant differences between PBS‐ and ACNPs‐treated mice, indicating that repeated ACNPs inhalation did not alter systemic RAS balance during long‐term administration (Figure 8L,M). Collectively, these findings demonstrate that repeated ACNPs administration three times weekly for 28 days caused no detectable systemic toxicity, pulmonary inflammation, fibrosis, immune activation, complement activation, or RAS imbalance. These results support the favorable long‐term biosafety of ACNPs.

## 3 Discussion

Conventional cell membrane‐based strategies, such as red blood cell membranes for prolonged circulation and macrophage membranes for inflammation targeting, have expanded biomedical applications, but remain limited by the intrinsic functions of native membranes [36, 37, 38]. Intelligent membrane engineering overcomes these constraints by enabling on‐demand customization of membrane proteins and chemical moieties through genetic engineering for high‐level protein expression or chemical insertion of functional groups, shifting the field from ‘structural mimicry’ to ‘functional programming’ [39, 40, 41, 42, 43].

In this study, we applied intelligent membrane engineering to construct ACNPs by integrating engineered cell membranes with high‐density ACE2 expression and chemically modified components. This design minimizes mucin adsorption, enhances ACE2‐mediated viral capture, and incorporates fusion inhibitors to intercept viral entry at its earliest stages. The modular ACNPs design creates a plug‐and‐play antiviral framework: by replacing displayed receptors, such as DPP4 for MERS‐CoV, CD4 for HIV, MXRA8 for chikungunya virus, or glycosaminoglycans for monkeypox virus, or by co‐expressing multiple receptors to generate pan‐viral nanosponge systems, the platform can be adapted to emerging pathogens. This programmable versatility redefines membrane‐based nanomedicine and offers a forward‐looking paradigm for broad‐spectrum, anticipatory antiviral defense [44].

The coronavirus disease (COVID‐19) pandemic and monkeypox outbreak have highlighted global vulnerability to emerging viruses, yet current antiviral strategies remain largely reactive. Potent neutralizing antibodies are constrained by rapid viral evolution, while remdesivir, nirmatrelvir, and tocilizumab act downstream by inhibiting viral replication, disrupting protease‐mediated polyprotein processing, and attenuating IL‐6–driven cytokine storms, respectively. Although these interventions can slow disease progression, they fail to prevent continuous viral entry or initial infection [45]. Accordingly, targeting early invasion events has emerged as a critical strategy for effective containment [46, 47, 48]. Conventional upstream approaches, including neutralizing antibodies, can block viral entry by targeting surface antigens and provide strong protection against specific strains; however, their efficacy and breadth are often limited by rapid viral evolution. Similarly, soluble ACE2 decoys can neutralize viral particles but often perform insufficiently under high viral burden or limited binding affinity [16, 17].

Herein, we redefine antiviral intervention by shifting from downstream inhibition to progressive upstream interception. ACNPs disrupt two essential viral entry steps, receptor engagement and membrane fusion, yielding a 22‐fold reduction in IC_50_ and suppressing pro‐inflammatory cascades. By severing the infection chain at its origin, active upstream blockade may reduce disease onset, address the limitations of late‐stage therapeutics, and mitigate downstream systemic injury. Compared with the human recombinant soluble ACE2 (hrsACE2), the representative ACE2‐based antiviral strategy that can neutralize SARS‐CoV‐2 at 200 µg/mL, ACNPs produced significant antiviral inhibition at much lower dose (containing 38 µg/mL ACNPs), owing to the combined effects of ACE2‐mediated viral sequestration and camostat‐mediated TMPRSS2 inhibition [14]. Beyond validation against SARS‐CoV‐2, this source‐level strategy establishes a broader paradigm for antiviral defense. By intercepting receptor recognition and membrane fusion at the earliest stage of invasion, ACNPs demonstrate scalable potential against diverse pathogens, including HIV, MERS‐CoV, chikungunya virus, and other high‐risk agents.

The platform is constructed from well‐established components, including phospholipids and PLGA, which underpin approved therapeutics and delivery systems, such as doxorubicin liposomes (Doxil/Caelyx) and risperidone microspheres (Risperdal Consta). These materials exhibit reliable safety profiles and undergo complete biodegradation into natural monomers, reducing concerns about long‐term accumulation and associated toxicity. In parallel, cell‐membrane–derived vesicles preserve native membrane proteins, and glycosylation patterns while circumventing the immune‐rejection and tumorigenicity risk associated with direct cell infusion. This multilayered safety architecture supports high biocompatibility, precise delivery, and a verifiable, regulatable, and scalable pathway for cross‐indication therapeutics and clinical translation.

Nevertheless, several limitations should be acknowledged. Extension of this platform to other viral systems would require the incorporation of corresponding host receptors or viral‐binding molecules. Moreover, longer‐term studies in larger animal models are needed to assess delayed immune responses, ACE2‐related physiological effects, nanoparticle clearance, and cumulative toxicity. Beyond material safety, the platform offers substantial flexibility in administration. Intranasal delivery enables targeted intervention at the primary sites of respiratory infection and can enhance early therapeutic efficacy. Its equipment‐free use reduces dependence on advanced infrastructure and specialized personnel, supporting deployment in household and community settings. During large‐scale outbreaks, this accessibility could enable rapid post‐exposure prophylaxis and early point‐of‐need intervention. By integrating therapeutic efficiency with deployability, the platform improves the patient experience and provides a practical direction for next‐generation broad‐spectrum antiviral defense.

## 4 Conclusion

We developed ACNPs that combine receptor customization, genetically engineered high‐density ACE2 display, and chemical tailoring to create a modular plug‐and‐play antiviral platform. Chemically tailored lipid components reduce mucin adsorption and improve mucus penetration, while surface‐presented ACE2 captures free virions and encapsulated camostat inhibits TMPRSS2‐mediated S protein cleavage and S2‐driven fusion, enabling early source‐level blockade. A single intratracheal dose achieved rapid broad‐spectrum antiviral activity, clearing more than 95% of SARS‐CoV‐2 variants, including wild‐type, Delta, and Omicron EG.5, within 72 h. Treatment achieved at least 80% survival against wild‐type SARS‐CoV‐2 and 100% survival against Delta and Omicron EG.5, while also potently inhibiting HCoV‐NL63. By coupling competitive neutralization with endocytosis blockade at the viral entry site, ACNPs provide a readily translatable modular platform for early intervention against current ACE2‐dependent coronaviruses and emerging “X viruses.”

## 5 Experimental Section/Methods

### 5.1 Materials

PLGA (lactide, glycolide (50:50); MW, 5000–15 000) were obtained from Sigma–Aldrich (USA). 1,2‐Distearoyl‐sn‐glycero‐3‐phosphoethanolamine‐N‐folate (poly(ethylene glycol) 2000 (DSPE‐PEG2000)) was purchased from Avanti (USA). Camostat mesilate was obtained from Selleck (USA). Hoechst 33258 and 3,3’‐dioctadecyloxacarbocyanine perchlorate (DiO) were obtained from Invitrogen (USA). Penicillin–streptomycin, DMEM/F12, trypsin–EDTA and fetal bovine serum were obtained from Gibco Life Technologies (USA). The mouse monoclonal antibody against ACE2 (Cat. No. sc‐390851) was purchased from Santa Cruz Biotechnology (USA). The rabbit polyclonal antibody against ATP1A1 (Cat. No. A01483) was obtained from GenScript (CHN). Rabbit polyclonal anti‐FLAG antibody (AF0036) was purchased from Beyotime Biotechnology (CHN). The rabbit polyclonal antibody against SV40 T antigen (Cat. No. PA5‐112037) was purchased from Invitrogen, Thermo Fisher Scientific (USA). A 220 nm polycarbonate membrane and Amicon ultra‐4 centrifugal filter with a molecular weight cut‐off of 10 kDa were purchased from Merck Millipore (GER).

### 5.2 Experimental models

Human embryonic kidney 293T cells, human lung adenocarcinoma Calu‐3 cells, and monkey kidney Vero‐E6 cells were obtained from the American Type Culture Collection (ATCC) or the Cell Bank, Chinese Academy of Sciences (CHN). DMEM medium was supplemented with 10% heat‐inactivated fetal bovine serum, 1% streptomycin, and 1% penicillin. Cells were cultivated at 37°C in a humidified atmosphere of 5% CO_2_. Female BALB/c mice (5–6 weeks old, 18–25 g) were purchased from Vital River Laboratory Animal Technology Co. Ltd., CHN. Human ACE2 transgenic mice (K18‐ACE2 mice, 5–8 weeks old, 20–25 g) were purchased from Cyagen Biosciences (CHN). The mice had access to food and water ad libitum and were hosted in ambient temperature (21‐26°C), humidity at 50%–60%, under 12 h dark/light cycles.

All the animal experiments were conducted following the guidelines of Chinese National Standard, and protocols were approved by the Animal Care and Use Committee at Shenzhen Third People's Hospital (approval number: 2024‐004). SARS‐CoV‐2 infection studies by authentic virus were conducted in the biosafety level‐3 (BSL‐3) laboratory of Shenzhen Third People's Hospital. All procedures were conducted in accordance with animal ethics guidelines and approved protocols to minimize suffering during vaccination, blood collection, and euthanasia.

### 5.3 Formulation and Characterization of ACNPs

Human ACE2 cDNA was cloned into the pCMV‐ACE2‐EGFP vector (Sino Biological, CHN) and transfected into HEK293T cells using Lipofectamine 3000 (Thermo Fisher, USA). The 293T cells were transduced for 12 h, then selected with 110 µg/mL hygromycin B for 7 days. Single‐cell clones were isolated by limiting dilution, expanded, and verified by ACE2‐GFP of confocal imaging. The 293T and ACE2‐293T cells were plated into eight‐well chambered coverglasses (Lab‐Tek, Nunc, USA). After being stained with DAPI, the cells were observed via confocal laser scanning microscopy (Leica SP8, GER). For flow cytometry analysis, 293T and ACE2‐293T cells were incubated with anti‐ACE2‐Alexa Fluor 647 (BioLegend, USA) for 30 min and washed with PBS three times. Flow cytometry (BD Accuri C6, USA) was used to analyse the Fluor 647‐positive ACE2‐293T cells. For western blot analysis, 293T and ACE2‐293T cells were lysed and quantified using a bicinchoninic acid (BCA) assay (Beyotime, CHN). Equal protein amounts were separated by 10% sodium dodecyl sulfate–polyacrylamide gel electrophoresis (SDS–PAGE), transferred to Polyvinylidene difluoride (PVDF) membranes, and blocked with 5% bovine serum albumin (BSA). Membranes were probed with anti‐ACE2 (Santa Cruz Biotechnology, USA), and anti‐GAPDH antibodies (Santa Cruz Biotechnology, USA) overnight at 4°C, followed by secondary antibodies. Signals were visualized using a chemiluminescence detection system (Bio‐rad, USA).

To extract ACE2‐293T cell membrane, ACE2‐293T cells (5 × 10^7^) were processed to remove intracellular contents through a combination of lysis and differential centrifugation (Beckman OptimaTM MAX‐XP Ultracentrifuge, USA). For formulation of ACNPs, camostat (2 mg) was dissolved in 1 mL of 4% ethanol, then emulsified with 1 mL Poly(lactic‐co‐glycolic acid) (PLGA) (2 mg/ml in acetonitrile) by sonication (39 W, 3 min) to produce drug‐loaded cores. Membrane vesicles were produced by co‐extruding ACE2‐293T cell membranes with DSPE‐PEG (180 µg) through 220 nm pores (Millipore, GER), then fused onto PLGA cores via 220 nm extrusion to give the ACNPs. The same procedures were used to prepare the ANPs without camostat. Nanoparticle size was measured by dynamic light scattering (Malvern, UK). To study the long‐term colloidal stability, ACNPs suspensions were adjusted to 1× PBS. Samples were stored at 37°C and the size of the ACNPs was measured once a day for 6 consecutive days. Morphology was imaged by TEM (FEI Tecnai G2 F20, USA). UV–vis spectra were recorded on a Lambda‐25 spectrophotometer (Perkin‐Elmer, USA). ACNPs were placed in 100 kDa dialysis bags and incubated in deionized water at 37°C with shaking (200 rpm). At predetermined time points, released camostat was quantified by high‐performance liquid chromatography (HPLC) (DIONEX ULTIMAT 3000, USA). For SDS‐PAGE, proteins were quantified by BCA assay, resolved on 10% SDS‐PAGE, and stained with Coomassie. For western blotting, samples were transferred to PVDF membranes, probed with anti‐ACE2 (Santa Cruz Biotechnology, USA), and anti‐ATP1A1 (GenScript, CHN), and then detected with secondary antibodies using chemiluminescence detection system (Bio‐Rad, USA). The binding strength between ACE2 and the receptor binding domain (RBD) of SARS‐CoV‐2 were investigated using the AutoDock Vina software (Scripps, USA). The three‐dimensional structures of ACE2 and the RBD of wildtype SARS‐CoV‐2, Delta, Omicron EG.5 were obtained from the Protein Data Bank (PDB: 6M17, 7WBQ, 8WRH) and incorporated into the docking workflow. To facilitate a comprehensive understanding of the ACE2‐RBD molecular complex, we utilized the PyMOL molecular visualization software (DeLano Scientific LLC, USA). PyMOL was used to generate high‐resolution graphical representations of the docked complex, enabling detailed examination of the intermolecular interactions and spatial arrangements of the key residues involved. Next, we performed MM‐GBSA binding free energy calculations on the trajectories, using the WT structure (PDB: 6M0J) as a unified template to prevent conformation‐induced resolution artifacts. To assess mucus penetration, artificial mucus was prepared by dissolving mucins (Sigma–Aldrich, GER) in aqueous solution. Samples were labeled with Cy7 and applied to the surface of the mucus layer. Fluorescence images were acquired at 0 and 3 h using an In vivo imaging system (IVIS system, USA) and analyzed with ImageJ to quantify penetration depth.

### 5.4 Assessment of ACNPs‐Mediated Viral Capture and Inhibition of Viral Entry In Vitro

Surface plasmon resonance (SPR) (Biacore T200, USA) assessed ACNPs binding to SARS‐CoV‐2 RBD. RBD (≈250 RU) was immobilized on a carboxymethyl dextran sensor chip (CM5, Cytiva, Denmark) via amine coupling (pH 5.0). ANPs or ACNPs were injected at in 4‐(2‐hydroxyethyl)‐1‐piperazineethanesulfonic acid (HEPES) buffer and data were fitted to a 1:1 Langmuir model. To assess viral capture of ACNPs, ACNPs were mixed with SARS‐CoV‐2 pseudovirus (37°C, 30 min), applied to carbon coated copper grid, and negatively stained with 1% phosphotungstic acid. Virus capture and nanoparticle morphology were visualised by TEM (FEI Tecnai G2 F20, USA). To evaluate S protein cleavage, Calu‐3 cells were co‐incubated with pseudoviruses and ACNPs for 2 h. Cell lysates were then analyzed by SDS–PAGE and Western blotting using an anti‐FLAG antibody (Beyotime, CHN) to detect S protein cleavage.

To assess antiviral ability of ACNPs against authentic SARS‐CoV‐2. SARS‐CoV‐2 (8 × 10^3^ PFU/ml) was pre‐incubated with serial dilutions of CNPs, ANPs or ACNPs (30 min, 37°C), then added to Calu‐3 cells. After 1 h adsorption, the inoculum was replaced with 1.6% carboxymethyl cellulose (CMC) overlay medium. Following 24 h incubation, cells were stained with anti‐SARS‐CoV‐2 antibody. Foci were developed with TrueBlue substrate and enumerated on an EliSpot reader (Agilent, USA). SPR sensorgrams were kinetically analyzed to obtain association (k_on_) and dissociation (k_off_) rates, with K_D_ calculated as k_off_/k_on_. Confocal imaging visual analysis of the recognition and viral entry blockade of ACNPs. Calu‐3 cells were pre‐seeded and infected with SARS‐CoV‐2 pseudovirus pre‐incubated with or without TCNPs, CNPs, ANPs, or ACNPs (37°C, 1 h). After 2 h, cells were stained with anti‐FLAG‐594 (BioLegend, USA) for virus labeling and 3,3’‐Dioctadecyloxacarbocyanine perchlorate (DiO) (Invitrogen, USA) for cell membrane labeling using CLSM imaging. Image J rendered 3D surface plots to quantify virus attachment and endocytosis. Luciferase activity was measured using d‐luciferin (Promega, USA) at 48 h to quantify relative viral load.

To distinguish the contribution of camostat‐mediated TMPRSS2 inhibition, Calu‐3 cells were treated with CNPs or ACNPs with an equivalent camostat concentration of 10 µm, followed by exposure to luciferase‐expressing SARS‐CoV‐2 pseudovirus. After infection, luciferase signals were detected using luciferase substrate (Revvity, Cat. 122799A) and quantified by the IVIS imaging system (Revvity, USA). To assess the ACE2‐mediated virus‐capture mechanism of ACNPs, ACNPs (containing 10 µm camostat, 1 mg/mL membrane protein) were pre‐incubated with S protein for 30 min to occupy the surface‐displayed ACE2‐binding sites and used as the reverse‐validation group (ACNPs + S protein). SARS‐CoV‐2 pseudovirus was then incubated with PBS, ANPs, ACNPs, or ACNPs + S protein at 37°C for 1 h before adding to Calu‐3 cells. Viral attachment and endocytosis were analyzed by confocal microscopy at 24 h, and pseudoviral load was quantified by luciferase assay at 48 h.

### 5.5 Inhibition of Pseudotyped SARS‐CoV‐2 (Wild‐Type, Delta, or Omicron) Infection In Vivo

BALB/c mice inhaled Cy7‐labeled ACNPs (50 µL, containing 5 mg/mL camostat, 6 mg/mL membrane protein) via MicroSprayer (BJ‐PW‐M, BioJane, CHN). Fluorescence signal of mice (740 nm excitation, 810 nm filter) was tracked by IVIS imaging system. At 72 h, organs were excised and imaged for biodistribution. To determine the minimum effective dose, K18‐hACE2 mice were challenged with pseudovirus (1 × 10^6^ TCID_50_/mL, 50 µL) and treated with five ACNP doses generated by threefold serial dilution from 15 mg/mL camostat and 18 mg/mL membrane protein. Antiviral efficacy was evaluated by IVIS imaging and lung immunofluorescence. For dosing optimization, pseudovirus‐challenged mice received either a single dose or three ACNPs doses at 24 h intervals, followed by pulmonary infection assessment. The therapeutic window was evaluated by administering ACNPs at 24, 48, or 72 h after challenge.

K18‐ACE2 mice inhaled luciferase‐tagged SARS‐CoV‐2 pseudovirus (1 × 10^6^ TCID_50_/mL, 50 µL) by MicroSprayer. 20 min later, mice received intratracheal nebulization of ANPs (50 µL, containing 6 mg/mL membrane protein), ACNPs (50 µL, containing 5 mg/mL camostat, 6 mg/mL membrane protein), or PBS (50 µL). Bioluminescence was quantified using an IVIS imaging system following administration of d‐luciferin (Promega kit, USA). At 72 h, lung sections were stained with anti‐ACE2‐FITC (Thermo Fisher, USA) to assess ACE2 expression. In addition, Anti‐Flag‐594 (BioLegend, USA) were performed to visualize SARS‐CoV‐2 pseudovirus in lung tissues using optical microscopy (Nikon, Japan). For pulmonary retention analysis, ACE2 and camostat levels were quantified in lung tissues at 0, 24, 48, 72, and 96 h after intratracheal ACNPs administration using ELISA and HPLC–MS, respectively.

To evaluate the infiltration of immune cells, the lungs were ground at 72 h and then filtered through a 100 µm cell strainer. The cell suspensions were stained for surface markers with anti‐CD45/CD3 and anti‐CD45/NK1.1 antibodies (BioLegend, USA), followed by analysis on a flow cytometer. Lung RNA was extracted with TRIzol reagent (Invitrogen, USA), reverse‐transcribed (Takara, Japan), and analyzed by quantitative polymerase chain reaction (qPCR) for IL‐1β, IL‐6, TNF‐α, and IFN‐γ. Gene expression was quantified using specific primers and normalized to Actin. Relative expression levels were calculated using the 2^−ΔΔCt^ method. Primers are listed in Table S2. At day 3 post‐inhalation, mice lungs were sectioned, and stained with hematoxylin and eosin (H&E) for histopathological evaluation. The pathological score was determined based on the INHAND Proposal (International Harmonization of Nomenclature and Diagnostic Criteria for Lesions in Rats and Mice). For RNA sequencing, mice lungs were harvested and RNA was isolated for RNA‐seq by Bio‐Pharm Technology (Majorbio, CHN) on the Illumina (NovaSeq XPlus) platform. Differential expression was analyzed using edgeR (corrected p < 0.05; fold change ≥ 2), and GO enrichment was performed using clusterProfiler (corrected p < 0.05). GSEA used GO, KEGG, and MSigDB gene sets (10–500 genes; p < 0.05), including Hallmark and C2 collections. PPI networks were constructed using STRING (combined score ≥ 400) and visualized with ggraph. For biosafety study, Mice inhaled a single high‐dose of ACNPs (50 µL, containing 15 mg/mL camostat, 18 mg/mL membrane protein). At day 3 post‐inhalation, mice lungs were sectioned, and stained with hematoxylin and eosin (H&E) for histopathological evaluation. Mice serum and whole blood were collected and hepatic function markers, renal function markers and multiple blood parameters were assessed using a Hitachi 7080 analyzer (Hitachi, Japan).

For long‐term safety evaluation, mice received ACNPs three times weekly for 28 days. Histological analysis, serum biochemical assays, immune response markers, complement activation, and RAS‐related factors were evaluated by H&E staining, Masson's trichrome staining, immunofluorescence, and ELISA. Residual dsDNA and SV40 large T antigen were quantified using fluorescence‐based assays and western blotting, respectively.

### 5.6 Inhibition of Authentic SARS‐CoV‐2 (Wild‐Type, Delta, or Omicron EG.5) and Authentic HCoV‐NL63 Infection In Vivo

K18‐ACE2 mice were intranasally challenged with 50 µL authentic SARS‐CoV‐2 (WT, Delta, Omicron 1 × 10^6^ TCID_50_/mL or HCoV‐NL63 1 × 10^5^ TCID_50_). 20 min later, mice received aerosolized administration of PBS (50 µL), ANPs (50 µL, 6 mg/mL membrane protein), or ACNPs (50 µL, 5 mg/mL camostat, 6 mg/mL membrane protein). At 3 days post‐infection, bronchoalveolar lavage fluid (BALF) was collected by flushing the lungs with 0.5 mL Hank's balanced salt solution (HBSS) through a needle inserted into the upper trachea. The recovered BALF was centrifuged at 15 000 × g for 15 min, and the supernatant was used for viral load quantification. Body weight and survival rate was monitored daily. Upon death or at day 14 post‐infection (for surviving animals), mice were euthanized at day 14. Lung tissues were collected for cytokine mRNA quantification and histological analysis. Lungs were sectioned and H&E‐stained for pathology.

### 5.7 Statistical Analysis

The data were subjected to statistical analysis using Prism 8.0 software (GraphPad, San Diego, CA). No data transformation or normalization was performed unless otherwise stated. All the data are reported as the mean ± standard deviation (SD), and the sample number (n) is specified in the corresponding figure legends. Data were analyzed using Student's t‐test or one‐way ANOVA followed by Tukey's post‐test. Here, ns indicates no significant difference, a single asterisk (*) indicates *p* < 0.05, a double asterisk (**) indicates *p* < 0.01 and a triple asterisk (***) indicates *p* < 0.001.

## Author Contributions

**Huimin Guo**: investigation, validation, methodology, funding acquisition. **Zhuojun He**: investigation, validation, methodology. **Yang Zhou**: writing – review and editing, visualization, resources, project administration, supervision. **Yang Zhang**: methodology, data curation, validation, investigation, project administration, writing – review and editing. **Bin Ju**: investigation, validation, visualization, writing – review and editing, formal analysis, supervision. **Min Shi**: conceptualization, investigation, validation, data curation, software, project administration. **Jian Ren**: validation, investigation, supervision, project administration. **Mingbin Zheng**: conceptualization, investigation, validation, formal analysis, supervision, resources, project administration, funding acquisition, writing – original draft, writing – review and editing. **Yeneng Dai**: investigation, validation, formal analysis, supervision, visualization. **Lanlan Liu**: supervision, resources, project administration, visualization, writing – review and editing. **Zhaozhen Li**: validation, formal analysis, supervision, project administration, visualization. **Pengfei Zhao**: funding acquisition, writing – original draft, writing – review and editing, supervision, resources, project administration, software, investigation. **Quan Fang**: validation, formal analysis, supervision, visualization, project administration. **Ze Chen**: methodology, investigation, validation, writing – original draft, project administration, writing – review and editing, conceptualization, data curation. **Ke Liu**: writing – review and editing, visualization, project administration, supervision, resources. **Qi Zhao**: investigation, conceptualization, validation, formal analysis, supervision, project administration, writing – review and editing. **Ruijing Liang**: supervision, resources, project administration, visualization, writing – review and editing, methodology. **Lintao Cai**: conceptualization, investigation, funding acquisition, writing – original draft, writing – review and editing, resources, supervision, formal analysis, project administration, visualization, software.

## Funding

National Key Research and Development Program of China (2021YFA0910900, 2023YFA0915600, 2023YFC2308300), Shenzhen Medical Research Fund (D2404002, D250402006), Shenzhen Science and Technology Program (KCXFZ20240903093903005, JCYJ20240813102021028, JCYJ20240813102012017), Natural Science Foundation of China (82372271), Key Area Projects for Universities in Guangdong Province (2022DZX2022), Guangdong Basic and Applied Basic Research Fund (2025A1515140076, 2025A1515010518), Shenzhen Clinical Medical Center for Emerging infectious diseases (LCYSSQ20220823091203007), and Shenzhen High‐level Hospital Construction Fund (23250G1005, 24250G1027).

## Conflicts of Interest

The authors declare no conflicts of interest.

## Supporting information




**Supporting File**: advs77592‐sup‐0001‐SuppMat.docx.

## Data Availability

The data that support the findings of this study are available on request from the corresponding author. The data are not publicly available due to privacy or ethical restrictions.
