# Supplementary material for: Intelligent Programmable Membrane Nanosponge for Early Virus Blocking
Source: Adv Sci (Weinh). 2026 Sep 16:e77592. Online ahead of print. doi: 10.1002/advs.77592 (PMC13583095; doi:10.1002/advs.77592)
Supplement: Supplementary file 1 [file ADVS-9999-e77592-s001.docx]

Supporting Information

Intelligent Programmable Membrane Nanosponge for Early Virus Blocking

Ze Chen, Zhuojun He, Huimin Guo, Min Shi, Ruijing Liang, Lanlan Liu, Yeneng Dai, Qi Zhao, Ke Liu, Yang Zhou, Yang Zhang, Jian Ren, Bin Ju, Quan Fang, Zhaozhen Li, Pengfei Zhao*, Mingbin Zheng*, Lintao Cai*

**Figure S1.** Size stability (A) and camostat release curve (B) of ACNPs.

**Figure S2.** Residual DNA and SV40 large T antigen content in ACNPs. (A) Residual DNA levels quantified using a fluorescence-based assay. Data are presented as mean ± SD (n = 3). (B) Western blot analysis of residual SV40 large T antigen in ACNPs. ATP1A1 is used as a membrane-protein loading control. Significance is calculated using one-way ANOVA with Tukey’s post-hoc test. *** *P* < 0.001.

**Figure S3**. Molecular dynamics (MD) simulation of ACE2–RBD (receptor binding domain of the S protein) binding interfaces. (A) Binding interfaces of ACE-2 and Spike protein RBD of Delta variants. purple: amino acid residues, yellow: RBD binding sites, red: mutated residues of Spike protein. (B) Binding interfaces of ACE-2 and Spike protein RBD of EG.5 variants. purple: amino acid residues, violet: RBD binding sites, red: mutated residues of Spike protein. Images were generated using PyMOL 3.0.3 software.

**Figure S4.** Visual and fluorescence inspection analysis of mucus penetration of ACNPs with DSPE-PEG (A) or without DSPE-PEG (B) through the artificial mucus layer. ACNPs functionalized with DSPE-PEG penetrated throughout the mucus layer, whereas ACNPs without DSPE-PEG remained largely confined to the upper region of artificial mucus layer.

**Figure S5**. Evaluating the antiviral activity of different nanoparticles against pseudovirus infection. (A) Quantitative analysis of pseudovirus inhibition efficiency of CNPs, TCNPs, ANPs and ACNPs. (B) Bioluminescence images of SARS-CoV-2 pseudovirus-infected Calu-3 cells after treatment with different nanoparticle formulations. Data are presented as mean ± SD (n = 3). Significance was calculated using one-way ANOVA with Tukey’s post-hoc test. *** *P* < 0.001, ns indicated not significant.

**Figure S6.** Pulmonary retention profiles of ACE2 and camostat following intratracheal administration of ACNPs. (A) ACE2 concentrations in lung homogenates at 0, 24, 48, 72, and 96 h after ACNPs administration, as determined by ELISA (n = 3). (B) Camostat content in lung tissue at the corresponding time points, as quantified by HPLC–MS (n = 3).

**Figure S7.** Quantitative analysis of wildtype pseudovirus in lung sections after treatments.

**Figure S8.** Dose-dependent antiviral efficacy of ACNPs against pseudovirus infection *in vivo*. (A) Representative whole-body bioluminescence images of pseudovirus-infected mice treated with five gradient doses of ACNPs, respectively. ACNPs containing 5 mg/mL camostat and 6 mg/mL membrane protein) are defined as C_1_. (B) Quantification of the average bioluminescence intensity in different groups (n = 3). (C) Representative fluorescence images of lung sections showing pseudovirus signals (red), ACE2 expression (green), and nuclei (blue). (D) Quantification of pseudovirus fluorescence intensity in the lung sections. Data are presented as mean ± SD (n = 3).

**Figure S9.** Inhibition of SARS-CoV-2 wild-type pseudovirus infection in ACE2-expressing mice by single-dose and three-dose ACNPs treatment. (A) Viral bioluminescence images in pseudovirus-infected ACE2 mice at 72 h post-treatment. ACNPs were administered once or every 24 h for three consecutive doses. (B) Quantification of retained viral bioluminescence *in vivo* after different treatments. (C) Immunofluorescence image of transverse lung sections. Viral antigen is shown in red, ACE2 in green, and nuclei in blue. scale bar, 50 μm. (D) Quantification of viral fluorescence intensity in lung sections after treatments. Data are presented as mean ± SD (n = 3). Significance is calculated using one-way ANOVA with Tukey’s post-hoc test. *** *P* < 0.001; ns indicates not significant.

**Figure S10**. *In vivo* biosafety of the multi-dose ACNPs treatment. (A) Representative H&E-stained sections of heart, liver, spleen, lung, and kidney after PBS or multi-dose ACNPs treatments. No obvious histopathological abnormalities were observed. Scale bars, 50 μm. (B–E) Evaluation of liver and kidney function indicators after multi-dose ACNPs treatment. Markers include ALT (alanine transaminase), AST (aspartate aminotransferase), UREA (blood urea nitrogen), and CREA (creatinine). Data are presented as mean ± SD (n = 3). Significance is determined using a two-sided unpaired Student’s t-test; ns indicates not significant.

**Figure S11**. Therapeutic activity of ACNPs against pulmonary pseudovirus infection 24, 48, or 72 h after pseudovirus challenge. (A) *In vivo* bioluminescence images of virus-infected K18-hACE2 mice treated with ACNPs 24 h, 48 h and 72 h after viral challenge. Mice were challenged with pseudotyped SARS-CoV-2. (B) Quantifying the bioluminescence intensities of pulmonary pseudovirus in the indicated groups. (C) Representative immunofluorescence images of lung tissue sections. Pseudovirus are shown in red, ACE2 in green, and nuclei in blue. (D) Quantifying the fluorescence intensities of pseudovirus in lung tissue sections. Scale bars, 100 μm.

**Figure S12.** ACNPs significantly inhibited Delta and Omicron pseudotype variant infection. (A) Virus bioluminescence images in Delta-infected ACE2 mice at 48 h and 72 h post treatment. (B) Quantitative bioluminescence intensities of retained virus *in vivo* after treatments. (C) Immunofluorescence imaging of transverse lung sections for Delta-infected ACE2 mice after ACNPs inhalation. Delta (red), ACE2 (green). (D) Quantitative analysis of Delta pseudovirus in lung sections. (E) Virus bioluminescence images in Omicron-infected ACE2 mice at 48 h and 72 h post treatment. (F) Quantitative bioluminescence intensities of retained virus *in vivo* after treatments. (G) Immunofluorescence imaging of transverse lung sections for Omicron-infected ACE2 mice after ACNPs inhalation. Omicron (red), ACE2 (green). (H) Quantitative analysis of Delta pseudovirus in lung sections. Data are presented as mean ± SD (n = 3). Significance is calculated using one-way ANOVA with Tukey’s post-hoc test. *** *P* < 0.001.

**Figure S13.** *In vivo* safety of ACNPs. (A) H&E staining of representative lung sections following tracheal atomization of ACNPs. (B-C) Evaluation of liver and kidney function indicators after ACNPs inhalation. Markers include ALT (alanine transaminase), AST (aspartate aminotransferase), ALP (alkaline phosphatase), CREA (creatinine), UA (uric acid), and UREA (blood urea nitrogen). (D-E) Complete blood count analysis in ACNP-treated mice. Parameters include WBC (white blood cells), RBC (red blood cells), PLT (platelets), Lymph (lymphocytes), Mon (monocytes), Gran (granulocytes), HCT (hematocrit), MCHC (mean corpuscular hemoglobin concentration), and MCV (mean corpuscular volume). Data are presented as mean ± SD (n = 3).

**Table S1**. Kinetic parameters derived from the SPR sensorgrams shown in Figure 3B. The values of k_on_, k_off_, and K_D_ were obtained by fitting the binding curves.

|  | k_on_ (M^-1^s^-1^) | k_off_ (s^-1^) | K_D_ (nM) |
| --- | --- | --- | --- |
| TCNPs | 3.54×10^6^ | 3.34×10^-2^ | 9.433 |
| ANPs | 3.61×10^7^ | 1.16×10^-2^ | 0.321 |
| ACNPs | 3.07×10^7^ | 9.92×10^-3^ | 0.323 |

**Table S2.** Primers used in quantitative real-time PCR (qRT-PCR).

| Gene | Species | Sequence | |
| --- | --- | --- | --- |
| SARS-CoV-2 | Human | (forward) | 5’- GGCAGTAACCAGAATGGAGAACG -3’ |
|  |  | (reverse) | 5’- ATGATGCCGTCTTTGTTAGCAC -3’ |
| HCoV-NL63 | Human | (forward) | 5’- AGGACCTTAAATTCAGACAACGTTCT -3’ |
|  |  | (reverse) | 5’- GATTACGTTTGCGATTAC CAAGACT -3’ |
| IL-1β | Mouse | (forward) | 5’- TCGTGCTGTCGGACCCATAT -3’ |
|  |  | (reverse) | 5’- GCCCAAGGCCACAGGTATTT -3’ |
| IL-6 | Mouse | (forward) | 5’- CACAAGTCCGGGAGGAGAC -3’ |
|  |  | (reverse) | 5’- TCCACGATTTCCCAGAGAAC -3’ |
| TNF-α | Mouse | (forward) | 5’- CCAAATGGCCTCCCTCTCAT -3’ |
|  |  | (reverse) | 5’- GGTGGTTTGCTACGACGTGG -3’ |
| IFN-γ | Mouse | (forward) | 5’- TGCCTGCTGGCAGATTGA -3’ |
|  |  | (reverse) | 5’- TGGGAAACCACAGTCCAGCT -3’ |
| Actin | Mouse | (forward) | 5’- GGAGATCACAGCTCTGGCT -3’ |
|  |  | (reverse) | 5’- GTCGATTGTCGTCCTGAGG -3’ |
